# Supplementary material for: A novel mechanism of streptomycin resistance in Yersinia pestis: Mutation in the rpsL gene
Source: PLoS Negl Trop Dis. 2021 Apr 22;15(4):e0009324. doi: 10.1371/journal.pntd.0009324 (PMC8096067; doi:10.1371/journal.pntd.0009324)
Supplement: S1 Table — (DOC) [file pntd.0009324.s001.doc]

S1 Table. Primers Used in this Study.

| **Primers** | **Nucleotide Sequence (5’ to 3’)** | **Product Size** |
| --- | --- | --- |
| YPO0199-DF1 | GCGctcgagCAACATGAAGCCGAGCAACTCA | 507 |
| YPO0199-DR1 | cgtgtaatgctgcaatctCATTATGAATACCCAAACCTC |
| Dcat-F | GAGGTTTGGGTATTCATAATGagattgcagcattacacg | 1135 |
| Dcat-R | CAGCATTACTCGCATTACCAtgtaacgcactgagaagc |
| YPO0199-DF2 | gcttctcagtgcgttacaTGGTAATGCGAGTAATGCTG | 495 |
| YPO0199-DR2 | GGactagtCGTACTTAGAACGTGATTG |
| Pwm91-F | GTTTTCCCAGTCACGAC | 232 (blank) |
| Pwm91-R | CAGGAAACAGCTATGAC |
| Cm-F | AGATTGCAGCATTACACG | 1135 |
| Cm-R | TGTAACGCACTGAGAAGC |
| strA-F | CTTGGTGATAACGGCAATTC | 348 |
| strA-R | AATGCCCAGTCGGCAGCG |
| strB-F | GGCGATTATAGCCGATCAAA | 198 |
| strB-R | GGATCGAGACAAAGGTCGTC |
| rpsL-F | TTCTGCGTCCTCGTTCTCTG | 489 |
| rpsL-R | TGTTTGGCCTTACTTAACGGAG |
| caf1-F | GGAACCACTAGCACATCTGTT | 249 |
| caf1-R | ACCTGCTGCAAGTTTACCGCC |

Note: underlined nucleotides indicate introduced restriction endonuclease recognition sites; lower case letters indicate overlapping regions.
