# Supplementary figures and images for: A novel mechanism of streptomycin resistance in Yersinia pestis: Mutation in the rpsL gene
Source: PLoS Negl Trop Dis. 2021 Apr 22;15(4):e0009324. doi: 10.1371/journal.pntd.0009324 (PMC8096067; doi:10.1371/journal.pntd.0009324)

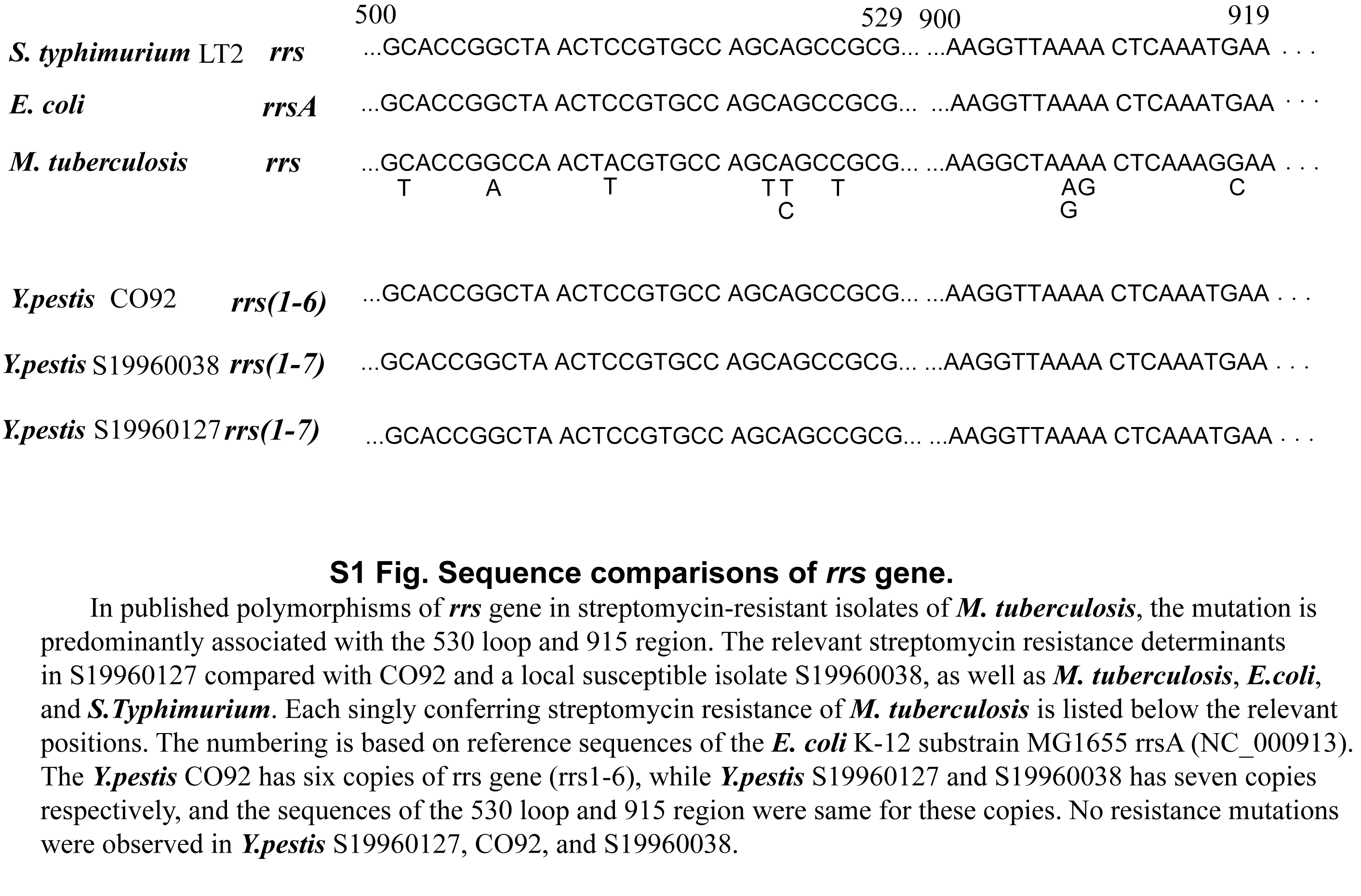

Supplement: S1 Fig — (TIF) [file pntd.0009324.s003.tif]
