# Supplementary material for: A novel mechanism of streptomycin resistance in Yersinia pestis: Mutation in the rpsL gene
Source: PLoS Negl Trop Dis. 2021 Apr 22;15(4):e0009324. doi: 10.1371/journal.pntd.0009324 (PMC8096067; doi:10.1371/journal.pntd.0009324)
Supplement: Attachment — Submitted filename: review_PNTD-D-20-01961_CS.docx [file pntd.0009324.s004.docx]

Dear Editor,

This manuscript entitled “A Novel Mechanism of Streptomycin Resistance in Yersinia pestis: Mutation in the rpsL gene” by Ruixia Dai *et al.* reports a non-described so far in *Yersinia* mutation in the *rpsL* gene associated with Streptomycin resistance.

Streptomycin is the reference treatment of plague, recommended by WHO. Other mutations, plasmid-mediated have already been described but this study reports the first mutation within a chromosomal gene responsible for Streptomycin resistance.

Overall this study is well conducted but I have some concerns that could be addressed by the authors before the manuscript be suitable for publication.

Concerns:

- I would add a conclusion sentence at the end of the abstract to highlight the impact of this study.

- L51-57: Please precise the genetic basis of the Antimicrobial resistance strains isolated in Madagascar.

- L60: Reference 6 corresponds to the description of a doxycycline resistant strain in Madagascar. The resistance being plasmid-mediated. Please add the corresponding reference for the Strain resistant in Mongolia and add a sentence regarding the doxycycline resistant strain isolated in Madagascar associated with reference 6.

- L67-74: To my point of view this part belongs to the result&discussion section.

- Results section: As the genome of several strains were sequenced I would suggest to use some easy-to-use bioinformatic tools to search for the presence of plasmid (as plasmidfinder or plasmidseeker) and to look for antimicrobial resistance genes (as Resfinder for instance).

- I Have a major concern regarding the Gene replacement of rpsL in the methodology. Could you include some modifications to make it easier to read? In the first step, could you include a description of the removal of the backbone of the plasmid for the mutant generation (like sucrose 10%? maybe). Maybe you should be a bit more clear by saying “to restore or to re-introduce the copy of the rpsL gene”.

- Nucleotide sequence accession numbers. In the manuscript, the genomes of 15 strains were sequenced but the accession number of only 1 strain (S1996127) is available. Please add the genome accession numbers for the other strains.

L211-213: Please show the alignment of ribosomal protein S12 and rrs in the figure 1 or state “data not shown”. But it would be preferable to see the alignment.

- In addition to the gene replacement experiment, your description is not sufficient to bring the evidence that the mutation is responsible for streptomycin resistance:

1. A complementation by restoring the rpsL mutation in the S1996127::rpsL-128A strain and evaluation of the MIC of Streptomycin would bring the ultimate evidence. Indeed, you cannot exclude that during the genetic manipulation, another mutation elsewhere in the genome was inserted (not on purpose) that led to the susceptible phenotype.

2. Another way to bring the evidence would be to sequence the genome of S1996127::rpsL-128A and compare it to S1996127 just to check that there is no mutation different from the one from rpsL gene.

3. The easier way would consist in testing other single clones from the step 2 of the gene replacement, just to make sure that independent clones have the same resistance phenotype.

- In the discussion, could you comment the treatment failure of Tetracycline and trimethoprim/sulfamethoxazole (as it was administered in combination with streptomycin) because the strain is not resistant to Tetracycline and trimethoprim/sulfamethoxazole.

- L257-259: Could you add data/comment/reference regarding Salmonella Streptomcyin resistant strain due to a mutation in *rpsL*.

- L270 – supplemental Table 3: could you precise how you place the strains in the phylogeny? It does not appear in the method section.

- L271 – 282: It is difficult to conclude that the mutation in rpsL doesn’t originate from local reservoir. Please be more cautious with your interpretation.

- L284- 293: It is not possible to state that the administration of streptomycin in patient B or C led to the emergence of streptomycin resistance. This resistance could have been present before the infection. Please reformulate.

- Significance of this research part. Could you add that monitoring of susceptibility to different antibiotics could be completed by genome sequencing and look for the presence of resistance genes of point mutation associated with resistance?

Minor comments:

L84: susceptibility to streptomycin. Please correct

L85-86: Please reformulate.

L88: Reference of CLSI should be more recent.

L91: Please precise “acceptable limits”

L95: Could you add more details regarding PCR conditions used?

L102: Supplemental Table 2. Please correct

L107 – supplemental Table 3: change the title as this table correspond to the sequenced strains.

L115: reference 15 does not refer to SOAP software nor de novo assembly. Please correct.

L159: Could you precise the MIC required for streptomycin resistance?

L186 L187: “Patient C”, “the strain”. Please remove the “s” at the end of “patient”and “strain”.

L196: the presence of a plasmid sharing 99.82% with pTP33 plasmid was identified. What about the coverage?

L197: Please replace “,” by “.” After (KT020860.1).

L268-270: Please reformulate.

L317: Please add “the” between “first” and “report”.
