# Supplementary material for: A novel mechanism of streptomycin resistance in Yersinia pestis: Mutation in the rpsL gene
Source: PLoS Negl Trop Dis. 2021 Apr 22;15(4):e0009324. doi: 10.1371/journal.pntd.0009324 (PMC8096067; doi:10.1371/journal.pntd.0009324)
Supplement: Attachment — Submitted filename: A letter containing a detailed list of responses -2021-2-11.doc [file pntd.0009324.s005.doc]

review_PNTD-D-20-01961

Notes : Dear Editors and reviewers，

Hereby, we responded all comments and suggestion in former reviewing letter point by point. Some problems we directly revised in manuscript. For those important issues we provided our answer or explain (blue word section) after the question, please see as follows, thank you.

Dear Dr. Li,
 
Thank you very much for submitting your manuscript "A Novel Mechanism of Streptomycin Resistance in Yersinia pestis: Mutation in the rpsL gene" for consideration at PLOS Neglected Tropical Diseases. As with all papers reviewed by the journal, your manuscript was reviewed by members of the editorial board and by several independent reviewers. The reviewers appreciated the attention to an important topic. Based on the reviews, we are likely to accept this manuscript for publication, providing that you modify the manuscript according to the review recommendations. 
 

This novel mechanism of Streptomycin resistance in Yersinia pestis is of interest for Public Health and deserves to be published. However the reviewers requested a series of precisions to clarify the experiments of gene replacement and some minor corrections.
Comments from the reviewer 1 are listed in an attached document.

 
Please prepare and submit your revised manuscript within 30 days. If you anticipate any delay, please let us know the expected resubmission date by replying to this email.  

When you are ready to resubmit, please upload the following:

[1] A letter containing a detailed list of your responses to all review comments, and a description of the changes you have made in the manuscript. 
Please note while forming your response, if your article is accepted, you may have the opportunity to make the peer review history publicly available. The record will include editor decision letters (with reviews) and your responses to reviewer comments. If eligible, we will contact you to opt in or out

[2] Two versions of the revised manuscript: one with either highlights or tracked changes denoting where the text has been changed; the other a clean version (uploaded as the manuscript file).

Important additional instructions are given below your reviewer comments. 

Thank you again for your submission to our journal. We hope that our editorial process has been constructive so far, and we welcome your feedback at any time. Please don't hesitate to contact us if you have any questions or comments.
 
Sincerely,

Anne-Sophie Le Guern
Guest Editor
PLOS Neglected Tropical Diseases

Javier Pizarro-Cerda
Deputy Editor
PLOS Neglected Tropical Diseases

***********************

Thank you very much for submitting your manuscript. 
This novel mechanism of Streptomycin resistance in Yersinia pestis is of interest for Public Health and deserves to be published. However the reviewers requested a series of precisions to clarify the experiments of gene replacement and some minor corrections.
Comments from the reviewer 1 are listed in an attached document.

Reviewer's Responses to Questions

**Key Review Criteria Required for Acceptance?**

As you describe the new analyses required for acceptance, please consider the following:

**Methods**

-Are the objectives of the study clearly articulated with a clear testable hypothesis stated?

-Is the study design appropriate to address the stated objectives?

-Is the population clearly described and appropriate for the hypothesis being tested?

-Is the sample size sufficient to ensure adequate power to address the hypothesis being tested?

-Were correct statistical analysis used to support conclusions?

-Are there concerns about ethical or regulatory requirements being met?

Reviewer #1: (No Response)

Reviewer #2: The methods appear sound.

Reviewer #3: The objectives of the study are clearly formulated with a clearly formulated testable hypothesis that the resistance of the clinical isolate of Yersinia pestis to streptomycin is determined by changes in its genome that occurred during the infectious / epidemic process during treatment with streptomycin.
The study design is appropriate to address the stated objectives.
The next questions are not applicable.

--------------------

**Results**


-Does the analysis presented match the analysis plan?

-Are the results clearly and completely presented?

-Are the figures (Tables, Images) of sufficient quality for clarity?

Reviewer #1: (No Response)

Reviewer #2: The results seem fine and support the conclusion that the K43R mutation in the S12 protein confers streptomycin resistance to Y. pestis.

Reviewer #3: The analysis presented match the analysis plan.
The results are clearly and completely presented.
The Tables & Figures are of sufficient quality for clarity.

--------------------

**Conclusions**


-Are the conclusions supported by the data presented?

-Are the limitations of analysis clearly described?

-Do the authors discuss how these data can be helpful to advance our understanding of the topic under study?

-Is public health relevance addressed?

Reviewer #1: (No Response)

Reviewer #2: The conclusions are reasonable.

Reviewer #3: Yes.
Not applicable.
Yes.
Yes.

--------------------

**Editorial and Data Presentation Modifications?**

Use this section for editorial suggestions as well as relatively minor modifications of existing data that would enhance clarity. If the only modifications needed are minor and/or editorial, you may wish to recommend “Minor Revision” or “Accept”. 

Reviewer #1: (No Response)

Reviewer #2: None required.

Reviewer #3: Lines 47-48: “… ANOTHER NEW BIOVAR, INTERMEDIUM, was proposed according to genetic diversity research [39]. Therefore, Y. pestis can be assigned into FIVE BIOVARS—Antiqua (glycerol positive, arabinose positive, and nitrate positive), Medievalis (glycerol positive, arabinose positive, and nitrate negative), Orientalis (glycerol negative, arabinose positive, and nitrate positive), Microtus (glycerol positive, arabinose negative, and nitrate negative), and Intermedium (its biochemical features still need to be determined) [38]”.

“… pestis [39].

From the MLVA analysis, A NEW BIOVAR, INTERMEDIUM, was proposed to describe rhamnose-positive Y. pestis subsp. pestis strains that occasionally infect humans and that are isolated mostly from marmots in the northern Tian Shan Mountains in China [39] … 
” [Qi Z, Cui Y, Zhang Q, Yang R. Taxonomy of Yersinia pestis. Adv Exp Med Biol. 2016;918:35-78. doi: 10.1007/978-94-024-0890-4_3].

**Answer: Thank you for your suggestion. We have added the corresponding nomenclature of the Biovars as your kind suggestion, and have added related literature in the revised version.**

Minor improvement in English required.
Restriction enzyme names have not been written in italics for a long time.

**Answer: As suggested, we have checked and corrected corresponding points in the revised version.**

Line 328: Serovar names do not need to be written in italics.

**Answer: As suggested, we have checked and corrected corresponding points in the revised version.**

Line 340: Is Table S1 really needed?
**Answer: Thank you very much for your comments and suggestions. Because in the “Methods ” section, we described a total of 536 *Y. pestis* isolates were used in this study. My original thought was give readers information about the strains we screened. Because in method section, we had presented clearly about the basic background of this strains in this study was isolated from 1946 to 2012 in 12 natural plague foci in China, so in order to avoid repeat, as you suggested, we deleted the Supplemental table1 in this revised version.**

--------------------

**Summary and General Comments**


Use this section to provide overall comments, discuss strengths/weaknesses of the study, novelty, significance, general execution and scholarship. You may also include additional comments for the author, including concerns about dual publication, research ethics, or publication ethics. If requesting major revision, please articulate the new experiments that are needed.

Reviewer #1: (No Response)

Reviewer #2: Dai et al. present a paper demonstrating a mutation in the rpsL gene of Y. pestis that confers high-level resistance to streptomycin. The paper as such is well written and the results convincingly demonstrate that the K43R mutation leads to streptomycin resistance. This apparently the first case of a chromosomal mutation reported for Y. pestis that increases resistance to streptomycin.This is of interest and medical significance.

However, I am not sure that the study goes far enough to warrant publication in PLoS Neglected Tropical Diseases. As the authors note in Figure 1, the same mutation is known to confer resistance in Enterobacteriaceae. To increase the impact of their study, the authors could conduct an evolution experiment with increasing to concentrations of streptomycin to find out how easily the rpsL mutation arises.

**Answer: Thank you very much for your kind comments and suggestions.**

**Just as you mentioned, in order to further clarify how easily for clinical isolate of *Y.pestis* develop a streptomycin resistance occurred during the infectious/epidemic process due to treatment of streptomycin. We are planning to conduct evolution experiments and** **competition experiments to find out mutations and fitness cost as your suggestions. We would like introduce related results in future. But as you know, all the experiments involving living *Y. pestis* must be performed in Biosafety Level 3 Laboratory, it will take a longer time to perform and get results. Streptomycin resistance of *Y. pestis* has great medical significance. In order to remind doctors and researchers the streptomycin resistance of *Y. pestis* as soon as possible, we hope this article will be published firstly.**

In addition, the authors should find out whether the mutation has any fitness costs by measuring growth curves or possibly performing in vivo competition assays against the wild-type version of the gene. If there are fitness costs, does the genome contain any potential compensatory mutations? What effects might these have?
**Answer: We totally agree with above reviewer’s suggestion, we are planning to perform the competition experiments to find out the effect of resistance on Growth. We would like to interpret this scientific question in future. As we mentioned above, in order to remind doctors and researchers the streptomycin resistance of *Y. pestis* as soon as possible, we hope this article will be published firstly.**

Reviewer #3: A team of Chinese scientists have prepared materials for publication that are especially relevant during the current pandemic. Their work once again reminds us that there are temporary difficulties and the problems that have accompanied man since prehistoric times. Adequate attention to these problems will save humanity from troubles that will make the current pandemic seem like a child's play. Overall, the manuscript is well written, but requires minor editing in English.
**Answer: thank you very much. We have revised the manuscript thoroughly according to your kind suggestion.**
--------------------

PLOS authors have the option to publish the peer review history of their article ([what does this mean?](https://journals.plos.org/plosntds/s/editorial-and-peer-review-process#loc-peer-review-history)). If published, this will include your full peer review and any attached files.

If you choose “no”, your identity will remain anonymous but your review may still be made public.

**Do you want your identity to be public for this peer review?** For information about this choice, including consent withdrawal, please see our [Privacy Policy](https://www.plos.org/privacy-policy).

Reviewer #1: No

Reviewer #2: No

Reviewer #3: Yes: Andrey P. Anisimov

Dear Editor,

This manuscript entitled “A Novel Mechanism of Streptomycin Resistance in Yersinia pestis: Mutation in the rpsL gene” by Ruixia Dai *et al.* reports a non-described so far in *Yersinia* mutation in the *rpsL* gene associated with Streptomycin resistance.

Streptomycin is the reference treatment of plague, recommended by WHO. Other mutations, plasmid-mediated have already been described but this study reports the first mutation within a chromosomal gene responsible for Streptomycin resistance.

Overall this study is well conducted but I have some concerns that could be addressed by the authors before the manuscript be suitable for publication.

Concerns:

- I would add a conclusion sentence at the end of the abstract to highlight the impact of this study.

**Answer: Thank you very much for your kind comments and suggestions.**

**In this revised version, we add a sentient to highlight the significance of this research, as follows:**

“Streptomycin is used as the first-line treatment against plague in many countries, and the emergence of resistance to streptomycin in *Y. pestis* represents a critical public health problem. So streptomycin susceptibility monitoring of *Y. pestis* isolates should not only include plasmid-mediated resistance but also include the ribosomal protein S12 gene (*rpsL*) mutation, especially when treatment failure is suspected due to antibiotic resistance.”

- L51-57: Please precise the genetic basis of the Antimicrobial resistance strains isolated in Madagascar.

**Answer: We have added the corresponding genetic basis related streptomycin resistance from Madagascar in manuscript, including descriptions about the conjugative plasmids in “Introduction” section.**

- L60: Reference 6 corresponds to the description of a doxycycline resistant strain in Madagascar. The resistance being plasmid-mediated. Please add the corresponding reference for the Strain resistant in Mongolia and add a sentence regarding the doxycycline resistant strain isolated in Madagascar associated with reference 6.

**Answer: We have added the corresponding description about the conjugative plasmids related streptomycin resistance from Madagascar in manuscript. Please see as follows. In addition, we also presented recently research result about doxycycline resistant strain isolated in Madagascar and cited corresponding literature in manuscript.**

Streptomycin is the most effective antibiotic agent against *Y. pestis* [4]. However, resistance to streptomycin has been reported in *Y. pestis* strains from Madagascar in two studies [5],[6]. In 1995, an isolate named 17/95 was isolated from a 16-year-old boy in the Ambalavao district of Madagascar and this strain exhibited multidrug-resistant traits to eight antimicrobial agents (streptomycin, chloramphenicol, tetracycline, sulfonamides, ampicillin, kanamycin, spectinomycin, and minocycline) [5]. The biovar of the strain 17/95 was orientalis and it carried the conjugative multidrug-resistant plasmid pIP1202 (a member of the IncA/C plasmid family), in which the high level of streptomycin resistance (MICs>2,048 mg/L) was due to the presence of streptomycin phosphotransferase activity induced by streptomycin resistance-associated genes (*strA* and *strB*) [5]. Another isolate named 16/95 (orientalis), only exhibiting streptomycin resistance, was obtained in 1995 in the Ampitana district of Madagascar from an axillary bubo puncture from a 14-year-old boy before antibiotic treatment [6]. The resistance determinant of 16/95 was carried by a self-transferable plasmid (pIP1203, a member of the IncP group) and the MIC of streptomycin for 16/95 was 1,024 mg/L. Recently, a paper reported plasmid-mediated doxycycline resistance in a *Y. pestis* strain in Madagascar (isolated from a rat in 1998) [7]. In addition, another multidrug-resistant *Y. pestis* strain was isolated in Mongolia in 2000 from a marmot[8], but the genetic basis and transferability of the resistance were not investigated [7].

**Still another, for the information that MDR *Y.pestis* strain was isolated from Mongolia in 2000, we firstly obtained the information in the literature 7. We looked back the literature, and found the piece of information originated from another paper (KieferD, DalantaiG, DamdindorjT, RiehmJM, TomasoH, ZollerL, etal. Phenotypical characterization of Mongolian *Yersinia pestis* strains. Vector Borne Zoonotic Dis 2012; 12:183–8.). We had added the paper in reference.**

- L67-74: To my point of view this part belongs to the result & discussion section.

**Answer: Thank you very much for your suggestions.**

**We revised corresponding sentences. And only left brief introduction about the purpose of research and the finding in this study. We wish such improvement could further meet the requirements in “Introduction” section.**

- Results section: As the genome of several strains were sequenced I would suggest to use some easy-to-use bioinformatic tools to search for the presence of plasmid (as plasmidfinder or plasmidseeker) and to look for antimicrobial resistance genes (as Resfinder for instance).

**Answer: Thank you very much for your kind suggestions, especially above mentioned softwares , which greatly facilitate our bioinformatic analysis. We had downloaded them and use them, and found that they are convenient, even it could told us the repeat number of plasmid. Thank you. In the near future, we will sequence more historical *Y.pestis* in China, we will fully considered above suggestion for more refined analysis.**

**Because this article had been basically formed, and we used to Resfinder：(**[**https://bitbucket.org/genomicepidemiology/resfinder/src/master/**](https://bitbucket.org/genomicepidemiology/resfinder/src/master/)**) to search the resistance associated with the presence of strA or strB present in mobile elements, but, no results could be found. Still, we used the Resfinder to search the point mutation, and no any feedback, we think it should be due to the mutation in the rpsL gene that encodes the ribosomal S12 protein: the mutation corresponded to a Lys to Arg substitution at site 43, is a our firstly found, so the date base did not record such mutation, even it is consistent with a similar mutation observed in Mycobacterium tuberculosis.**

**So we only added a sentence to illustrate that we used the plasmidfinder in “Method” section (https://bitbucket.org/genomicepidemiology/plasmidfinder/src/master/) to analysis the characteristics of plasmids, for the reason that latter part of manuscript we used the software identify the coverage and identity rate of pS96127 compare to pTP33.**

**We had added the corresponding reference in manuscript.**

- I Have a major concern regarding the Gene replacement of rpsL in the methodology. Could you include some modifications to make it easier to read? In the first step, could you include a description of the removal of the backbone of the plasmid for the mutant generation (like sucrose 10%? maybe). Maybe you should be a bit more clear by saying “to restore or to re-introduce the copy of the rpsL gene”.

**Answer: Thank you very much for your comments and suggestions.**

**We rewrote corresponding sentence or words to make it easier to read, including adding some sentence before experimental process to describe the purpose, just like you suggested. Please see corresponding description in revised manuscript. We wish such improvement could make our description more easy to read.**

- Nucleotide sequence accession numbers. In the manuscript, the genomes of 15 strains were sequenced but the accession number of only 1 strain (S19960127) is available. Please add the genome accession numbers for the other strains.

**Answer: As suggested, we have provided the whole-genome information, including the accession number code deposited in the NCBI GenBank, in revised Supplement Table 2.**

**Another apologetic question is that, for the some reason, there was a “0” different between the strains named in first version of supplemental files 3 (this version is file 2) and the strains names we deposited in NCBI GenBank. In this revised version, we revised them in supplemental table 2 in accordance with NCBI GenBank information, and we checked the manuscript thoroughly and corrected all such difference.**

L211-213: Please show the alignment of ribosomal protein S12 and rrs in the figure 1 or state “data not shown”. But it would be preferable to see the alignment.

**Answer: Thank you very much for your suggestions.**

**Sequence comparisons of RpsL protein was illustrated in Figure 1. And such comparison could present the mutation in the rpsL gene that encodes the ribosomal S12 protein: the mutation corresponded to a Lys to Arg substitution at site 43, and is consistent with a similar mutation observed in M. tuberculosis, et.al. While, there were no corresponding mutation in the streptomycin susceptible strain CO92 and S19960038. In addition, the Sequence comparison of *rpsL* gene showed no amino-acid alteration at position 88 of ribosomal protein S12 in S19960127 (figure 1).**

**For the rrs mutations in streptomycin-resistant isolates of M. tuberculosis, the mutations in rrs are associated with a low or intermediate level of resistance (tolerating streptomycin at concentrations between 50 and 500 mg/L) in M. tuberculosis, while, in this study, high-level resistance to Streptomycin in Y. pestis from pneumonic plague outbreak was identified.**

**In addition, for the rrs mutations in streptomycin-resistant isolates of M. tuberculosis, the mutation is predominantly associated with the 530 loop and 915 region in secondary structure of 16SrRNA; Just as suggested, we compared the sequences in 530 loop and 915 region of rrs gene and illustrated in supplemental figure1. In which we referenced the literature 19 (Dal Molin M, Gut M, Rominski A, Haldimann K, Becker K, Sander P. Molecular Mechanisms of Intrinsic Streptomycin Resistance in Mycobacterium abscessus. Antimicrob Agents Chemother. 2018;62(1). Epub 2017/10/25. doi: 10.1128/AAC.01427-17. PubMed PMID: 29061744; PubMed Central PMCID: PMCPMC5740355.) , and the literature 20. In these literatures, the alignments of rpsL and rrs were illustrated. And we listed above literatures in reference.**

**The *Y.pestis* CO92 has six copies of rrs gene (rrs1-6), while the *Y.pestis* S19960127 and S19960038 has seven copies(rrs1-7) respectively, and the sequences the 530 loop and 915 region were same for these copies. No resistance mutations were observed in *Y. pestis* S19960127, CO92, and S19960038.**

- In addition to the gene replacement experiment, your description is not sufficient to bring the evidence that the mutation is responsible for streptomycin resistance:

1. A complementation by restoring the rpsL mutation in the S19960127::rpsL-128A strain and evaluation of the MIC of Streptomycin would bring the ultimate evidence. Indeed, you cannot exclude that during the genetic manipulation, another mutation elsewhere in the genome was inserted (not on purpose) that led to the susceptible phenotype.

**Answer: Thank you very much for your comments and suggestions.**

**Firstly, we would like to say that, it is in the proceeding of screening multi-antibiotics susceptibility in *Y.pestis* in China, we found the high-level resistance to Streptomycin in *Yersinia pestis* and then found that it was associated with a Pneumonic Plague Outbreak in Tibet, then we used the PCR of strA and strB to identify the target, however obtained negative results, which arouse us to sequence the whole genome of the strains and found the mutation of rpsL gene could explain the reason, following allelic single-nucleotide polymorphism (SNP) replacement experiments in vitro demonstrated above inference.**

**Secondly, the Allele exchange was carried out according to previous literature** (Zhou YY, Zhang HZ, Liang WL, Zhang LJ, Zhu J, Kan B. Plasticity of regulation of mannitol phosphotransferase system operon by CRP-cAMP complex in Vibrio cholerae. Biomed Environ Sci. 2013;26(10):831-40. Epub 2013/11/13. doi: 10.3967/bes2013.006. PubMed PMID: 24215877.), **and the author team in this paper worked with our team in same institute in China CDC. We consulted them and used corresponding gene manipulation facilities and tool plasmids (pWM91, et.al ) and we also listed one member in above paper in our manuscript. Hence, we would like to say the allele exchange operation platform should be mature, and in every step of gene manipulation, we used the target PCR to confirm and screen the MICs of associated strain in intermediate process. In addition, All the experiments associated with Y. pestis, as well as key replacement experiments, all were conducted in the Bio-safety Level 3 (BSL-3) laboratory. The BSL-3 was a newly-built laboratory and special to performer corresponding experiment for Y.pestis. So we would like to say, we have reasonable confidence there were very limited possibility brought into target gene or point mutation by chance through un-intentional wrong operation.**

**Thirdly, and most important, in our allele exchange assay, we need change the phenotype with Streptomycin resistance to sensivitic Streptomycin phenotype, we adopted massive screening strategy. In the step that the E. coli SM10λpir with recombinant plasmids combined with S19960127△YPO0199::cat, The massive growing colonies were screenned by PCR and MIC method. In fact , at the beginning, we used the Luria–Bertani (LB) plates to cultured these mixtures, and failed even though we put into amount of works. Later we used the violet agar plates to incubate these mixtures and obtained correct mutation of *Y.pestis*. And then the rpsL mutant (Y. pestis S19960127:: rpsL-128A) was transferred to 10% sucrose (W/V) plates at 22℃ for 36–48 h to remove the plasmid backbone Pwm91. After the above gene operation, we consider there should exist little chance to cause another mutation elsewhere in the genome was inserted, for only above mentioned factors involved in the expriment and the operation was performed in BSL-3 laboratory.**

**Above all , We wish above explanations could eliminate corresponding doubt .**

2. Another way to bring the evidence would be to sequence the genome of S19960127::rpsL-128A and compare it to S19960127 just to check that there is no mutation different from the one from rpsL gene.

**Answer: Thank you very much for your comments and suggestions.**

**We fully agree that the whole genomic sequencing should be one of way to confirm the mutation at 128 bp (G-A) that could be attributed to the forming of streptomycin resistance. For some reasons, so far, we have some difficult to further whole sequencing genome of S19960127::rpsL-128A, not only on financial reason, mainly because of the facility of BSL-3 which were not available up to now. So In the future, we could sequence the genome of strains and comparing corresponding point.**

**In another side, genome sequencing also could bring some new problem, such as new culture or some other reason could bring point mutation or gene deletion on genome of sequenced strains. At this situation, there would present a dilemma, could we prove such mutation originated from culturing or form other reasons, which bringing the changes could be proved no contributed to streptomycin resistance or sensitivity. So there should be an unending circle. Even though genome sequencing could let us become more and more confident that rpsL-128A mutation is the reason. However, in another side, we could not completely rule out above mentioned possibility. Hereby, please do not consider the mutation or error is just like the situation mentioned in above term, i.e. target gene or point mutation of rpsL were introduced by chance through un-intentional wrong operation. On one side, we used the PCR to screen periodically, for the other the rang and point occurred should also not be same.**

3. The easier way would consist in testing other single clones from the step 2 of the gene replacement, just to make sure that independent clones have the same resistance phenotype.

**Answer: thank you for your suggestion, in order to identity the issue of streptomycin resistance replacement on rpsL-128 G-A was the real reason, in fact, we did some verification and evaluation work on every step, we would like elaborate as follows:**

**In one way, we did checking in every step of gene replacement, by screening the specific gene target by PCR (note, in this revised manuscript, we also added the primers of calf1 gene that we used to confirm the target bacterium was *Y. pestis* in supplemental table 2, the step was in fact periodically performed in our experiment );**

**In another way, we checked the MIC of streptomycin on every step. So it was reasonable to ensure that it was the mutation at no. 128 bp allele of rpsL gene was the reason for exchange of streptomycin resistance. Just as mentioned, the replacement of the rpsL gene with the mutant allele (G128-A) was confirmed by PCR (primers: rpsL-F/rpsL-R) and DNA sequencing.**

**In order to further illustrate above proceeding, we added the MIC values of intermediate engineered strains in table 2. Please see in table 2. We wish such improvement could make our description more clearly and could eliminate your corresponding doubt.**

- In the discussion, could you comment the treatment failure of Tetracycline and trimethoprim/sulfamethoxazole (as it was administered in combination with streptomycin) because the strain is not resistant to Tetracycline and trimethoprim/sulfamethoxazole.

**Answer: Thank you very much for your suggestions.**

**We totally agree that it is very important to discuss the treatment failure in this pneumonic plague outbreak that caused four patients and four all died, even though the combination treatment were administrated including the streptomycin.**

**We have added corresponding sentences to mention this issue in this revised manuscript and hereby, we listed possible reasons one by one.**

**Streptomycin is the most effective antibiotic against *Y. pestis* and the drug of choice for treatment of plague, particularly for the pneumonic form (Plague Manual: Epidemiology, Distribution, Surveillance and Control: WHO/CDS/CSR/EDC/99.2). Of course, Streptomycin, chloramphenicol, and tetracycline are used to treat plague, and tetracycline and sulfamethoxazole are recommended for prophylaxis.**

**On one hand, one of possibility about the treatment failure was due to streptomycin resistance, while other antibiotics, such as tetracycline and sulfamethoxazole, comparatively, are not as effective as streptomycin, especially for the pneumonic plague. Plague Manual published by WHO also mentioned that some studies have shown higher mortality, increased complications, and longer duration of fever when only Sulfonamides was used.**

**On the other hand, it maybe also due to severity of the disease and delayed treatment. The plague is a severe disease with high fatality rate, so we think treatment failure of plague were also likely due to the severity of the disease, especially in pathogen Y. pestis with biovar antique. T**he *M. himalayana* plague focus on the Qinghai-Tibet plateau is the largest and most active focus in China. In this natural plague focus, the pathogen *Y. pestis* (biovar antiqua) frequently causes pneumonic or septicemic plague with high mortality (we have literature to illustrate it). **For example, the national plague surveillance reports in China show that a total of twenty-two human plague outbreak evens, involving in 120 human plague cases with 75 deaths in Tibet were reported during 1966 and 2010.**

- L257-259: Could you add data/comment/reference regarding Salmonell a Streptomcy in resistant strain due to a mutation in *rpsL*.

**Answer: As suggested, we have added related literature in the revised version.**

- L270 – supplemental Table 3: could you precise how you place the strains in the phylogeny? It does not appear in the method section.

**Answer: Thank you very much for your comments and suggestions.**

**As far as the methods associated with reconstructing the phylogenetic relationship and corresponding algorithm concerned in this study, we mainly used the methods in the paper of Giovanna Morelli (Giovanna Morelli，et al,** **Y*ersinia pestis* genome sequencing identifies patterns of global phylogenetic diversity.** [**Nat Genet.**](http://www.ncbi.nlm.nih.gov/pubmed/?term=Yersinia+pestis+genome+sequencing+identifies+patterns+of+global+phylogenetic+diversity) **2010. 42(12):1140-3) and the paper of Cui.Y,et al,( Historical variations in mutation rate in an epidemic pathogen, *Yersinia pestis*. Proc Natl Acad Sci USA. 2013 Jan 8;110(2):577-82. ) to construct the phylogenetic relationship tree.**

**Referring previously sequence data and the nomenclature of phylogenetic lineages in above literatures. For strains in this study, if they clustered in a lineage, while coincidently, there were one or more known strains that previously be designated nomenclature in certain lineage. The nomenclature of the strain studied will be given same nomenclature. If the strain did not fall in any designated nomenclature, so we annotated the strain only presented the population name in phylogenetic branch.**

**We have added corresponding description in note in supplemental table 3s.**

- L271 – 282: It is difficult to conclude that the mutation in rpsL doesn’t originate from local reservoir. Please be more cautious with your interpretation.

**Answer: Thank you very much for your comments and suggestions.**

**In our study, based on historical *Y. pestis* strains collection, we found that The S1996127 strain isolated from Qayü village, Lhünze County clustered into the 2.ANT2d** **lineage in the phylogenetic tree. The same lineage group also included Y. pestis S1996038 (2.ANT2d), the strain isolated from a patient in Cona County in 1996. In addition, other isolates from nearby regions in Lhünze County and Cona County were also included in the 2.ANT2d lineage. Such results indicate that one *Y. pestis* clone is persistently present in host animals and humans and causes various epidemics events. However, all these strains did not exhibited substitution mutation in rpsL gene. So we summed such conclusion that the high level of resistance by a mutation did not originate from the local reservoir of *M. himalayana* or previous plague outbreaks.**

**In order to leave leeway for the conclusion, we would like to consider tone down corresponding statement. So we rewrote the sentence and used the word “inferred” to suggest that there were such a possibility.**

- L284- 293: It is not possible to state that the administration of streptomycin in patient B or C led to the emergence of streptomycin resistance. This resistance could have been present before the infection. Please reformulate.

**Answer: Thank you very much for your comments and suggestions.**

**We had reformulated corresponding statement, just like you suggested.**

**In one side, just like above mentioned, the *Y. pestis* strains isolated from local reservoirs of *M. himalayana* or previous plague outbreaks all did not exhibited substitution mutation in rpsL gene, and the evaluation of streptomycin resistance indicated they all belonged to sensitive clone.**

**Another reason is in the *M. himalayana* focus on the Qinghai-Tibet plateau in China. for the hosts of animal plague were *M. himalayana*, which inhabited in a comparatively “clearer” circumstance, another way of saying is there was few influence from human being, so the background of streptomycin should be very low.**

**Most importantly, according to epidemiologic investigation and the treatment records of clinics ‘( please see corresponding section in result), the patient C or patient B had been administrated streptomycin, with other combined antibiotics, however they were not cured. (of course the treatment failure were likely attribute to the severity of pneumonic plague disease, we also mentioned the possibility in manuscript). so we inferred that the resistance exchange should associated with the treatment of streptomycin.**

**Similarly, in order to leave leeway for the conclusion, we tone down corresponding statement and used the sentence: “So the emergence of streptomycin resistance was suspected due to the streptomycin application in Patient C or her predecessor (Patient B).”**

**And, in the late section of this paragraph, we added a sentence “Of course, Additional studies investigating the origination of streptomycin resistance are still needed *in vitro* or *in vivo* to prove such selective function”** **for this purpose of tone down above conclusion statement.**

- Significance of this research part. Could you add that monitoring of susceptibility to different antibiotics could be completed by genome sequencing and look for the presence of resistance genes of point mutation associated with resistance?

**Answer: Thank you very much for your comments and suggestions.**

**We have added the corresponding description about the usefulness of whole genome sequencing approaches to detect those known and potentially novel mutations associated with drug resistance. As follows:**

“Currently, genome sequencing became significantly easier with the advance of next generation sequencing technologies. On one hand, the genome-wide SNP analysis could be used to illustrate the phylogenetic relationship or the microevolution of *Y. pesti*s, including used for source-tracking in plague outbreaks. On the other hand, whole genome sequencing and associated analysis methods could greatly facilitate the detection of known and potentially novel mutations associated with antibiotics resistance in *Y. pesti*s. Ultimately, such insights will greatly assist with patient treatment, management, and the disease control.”

Minor comments:

L84: susceptibility to streptomycin. Please correct

L85-86: Please reformulate.

**Answer: we have exchanged original inappropriate literature and rewrote the sentence to make it more clearly and more coherent. Please see in revised manuscript.**

L88: Reference of CLSI should be more recent.

**Answer: We have replaced the corresponding reference with updated standard issued by Clinical and Laboratory Standards Institute (CLSI), corresponding literature was referenced in revised manuscript.**

L91: Please precise “acceptable limits”

**Answer: we rewrote the sentence.**

**i.e. “**Quality control strains (*Escherichia coli* ATCC 25922 and *Pseudomonas aeruginosa* ATCC27853) were tested periodically with each batch of *Y. pestis* isolates to validate the accuracy of the procedure”.

L95: Could you add more details regarding PCR conditions used?

**Answer: We have added the details of PCR experiment in “Methods”, please see in revised manuscript.**

L102: Supplemental Table 2. Please correct

L107 – supplemental Table 3: change the title as this table correspond to the sequenced strains.

**Answer: We have changed the title of supplemental table and illustrated the table correspond to the sequenced strains**

L115: reference 15 does not refer to SOAP software nor de novo assembly. Please correct.

**Answer: Thank you very much for your comments and suggestions.**

**Sorry for our mistakes, we have changed corresponding reference literature in this revised version.**

L159: Could you precise the MIC required for streptomycin resistance?

**Answer: sorry, I can’t accurately catch your meaning bout “MIC required for streptomycin resistance”, do your meaning is the susceptibility breakpoints established for *Y. pestis*?**

**I added corresponding description as follow:”the MIC breakpoint of streptomycin resistance stipulated by CLSI is ≥16 mg/L” in revised manuscript. Meanwhile gave corresponding literature.**

L186 L187: “Patient C”, “the strain”. Please remove the “s” at the end of “patient”and “strain”.

L196: the presence of a plasmid sharing 99.82% with pTP33 plasmid was identified. What about the coverage?

**Answer: we used the plasmidfinde software to identify the coverage and identity rate of pS96127 compare to pTP33 is 99.98 and 99.95 respectively. We have added corresponding description in this revised version.**

L197: Please replace “,” by “.” After (KT020860.1).

L268-270: Please reformulate.

**Answer: we rewrote the sentence to make it more clearly and more coherent. As follows**

The epidemiological investigation indicated that the Patient C took care of Patient B (primary pneumonic plague) and unfortunately got infection. Considering corresponding clinical symptoms and treatment of patients, as well as the genomic phylogenetic relationship in associated strains (molecular subpopulation designations see in supplemental Table 2), we inferred that the high level of resistance by a mutation did not originate from the local reservoir of *M. himalayana* or previous plague outbreaks,

L317: Please add “the” between “first” and “report”.

Figure Files:
While revising your submission, please upload your figure files to the Preflight Analysis and Conversion Engine (PACE) digital diagnostic tool, https://pacev2.apexcovantage.com. PACE helps ensure that figures meet PLOS requirements. To use PACE, you must first register as a user. Then, login and navigate to the UPLOAD tab, where you will find detailed instructions on how to use the tool. If you encounter any issues or have any questions when using PACE, please email us at figures@plos.org.
  **Answer: We have checked our revised** **figure 1 and 2 in manuscript using the PACE tool to help us ensure the figures meet PLOS requirements. PACE tools pass above query.**

Data Requirements:
Please note that, as a condition of publication, PLOS' data policy requires that you make available all data used to draw the conclusions outlined in your manuscript. Data must be deposited in an appropriate repository, included within the body of the manuscript, or uploaded as supporting information. This includes all numerical values that were used to generate graphs, histograms etc.. For an example see here: http://www.plosbiology.org/article/info%3Adoi%2F10.1371%2Fjournal.pbio.1001908#s5.
 
Reproducibility:
To enhance the reproducibility of your results, PLOS recommends that you deposit laboratory protocols in protocols.io, where a protocol can be assigned its own identifier (DOI) such that it can be cited independently in the future. For instructions see http://journals.plos.org/plosntds/s/submission-guidelines#loc-materials-and-methods

__________________________________________________
In compliance with data protection regulations, you may request that we remove your personal registration details at any time. (Use the following URL: https://www.editorialmanager.com/pntd/login.asp?a=r). Please contact the publication office if you have any questions.

Manuscript ID: PNTD-D-20-01961R1
The letter provided on 1st, Feb, 2021 from editors

Dear Dr Li,

Thank you for submitting your paper, "A Novel Mechanism of Streptomycin Resistance in Yersinia pestis: Mutation in the rpsL gene", to PLOS Neglected Tropical Diseases. We have checked over your submission and, before we can assign your manuscript to the Editor, we ask that you first please make the following modifications:

1) We note that the following authors have not yet confirmed their authorship on this manuscript: Yumeng Wang, Jianguo Xu.

At this point this request is not urgent; we have re-sent their co-author confirmation email containing an auto-confirmation link for them to click, and we will reach out to them at a later stage if we don't hear from them. We wanted to mention this in case you are aware of them experiencing technical issues with confirming authorship via the link. If so, please do ask them to contact us directly via email and we'd be happy to assist. 
 **Answer: We have asked Prof. Jianguo Xu, and Dr. Yumeng Wang to confirmed their authorship on this manuscript.**

2) Please provide an Author Summary. This should appear in your manuscript between the Abstract (if applicable) and the Introduction, and should be 150–200 words long. The aim should be to make your findings accessible to a wide audience that includes both scientists and non-scientists. Sample summaries can be found on our website under Submission Guidelines.
**Answer: We have attached an Author Summary (Synopsis) in the corresponding site in this revised manuscript.**

3) We noticed that you used “data not shown” in the manuscript, we do not allow these references. The PLOS data access policy requires that all data be either published with the manuscript or made available in a publicly accessible database. Please amend the supplementary material to include the referenced data or remove the references. Please make a note of this change in your response to reviewers.
**Answer: There were a total of four sites that we used the “data not shown”, we responded here together, as follows, and hope reviewer’s kind understanding,**

1, In “Apart from S19960127, another 14 strains sensitive to streptomycin isolated in Shannan Prefecture also carried the pS96127 plasmid (data not shown).”

**Besides the S19960127, the sequences of other total 14 strains we also had deposited in NCBI GenBank. In this revised version, we listed them in supplemental table 2 in accordance with NCBI GenBank information, so here the pS96127 plasmid sequences could be found in these sequences. So we directly delete the note hereby.**

2, “This SNP point mutation was confirmed by PCR amplification and Sanger sequencing (data not shown).”

**Here, we would like to present the way that we used to identify the mutation of the rpsL gene, because such PCR and PCR products sequencing is a common common technology and it belong to method description, so we think it is more appropriate to move the sentence to the method section , in addition, we deleted the note ”data not shown” in method section.**

3, in “results” section, “In addition, no amino-acid alteration at position 88 of ribosomal protein S12 or *rrs* gene mutations in S19960127 were found (data not shown)”

**Just as the suggestion given from reviewer 1, we illustrated the alignments of rrs in supplemental figure1. In this result, among the *Y.pestis* species, by comparing, those sequenced strains in our study and in previously published sequenced strains, no any difference on the 530 loop and 915 region.**

**In addition, the alignments of amino-acid alteration at position 88 of ribosomal protein S12 could be found in figure1. Hence, we added corresponding note in labeling reference in figure 1 (position 88 of ribosomal protein S12) and *rrs* gnen (supplemental figure1).**

4， In Discussion section. “Sequence comparison showed no amino-acid alteration at position 88 of ribosomal protein S12 or *rrs* gene mutations in S19960127 (data not shown).”

**The answer is same with above.**

4) Tables should not be uploaded as individual files. Please remove this file (Table 1. The Strains and Plasmids-2021-1-29.docx) and include the table in your manuscript file.

5) Please amend your detailed Financial Disclosure statement. This is published with the article, therefore should be completed in full sentences and contain the exact wording you wish to be published.

Please include all sources of funding (financial or material support) for your study. List the grants (with grant number) or organizations (with url) that supported your study, including funding received from your institution. 

State the initials, alongside each funding source, of each author to receive each grant.

Please ensure you include your funder's role statement "The funders had no role in study design, data collection and analysis, decision to publish, or preparation of the manuscript." at the end of your amended statement.

Once you have made the necessary changes in Editorial Manager, please re-approve the submission by following the same steps as before.

If you have any questions or require assistance, please don't hesitate to get in touch. To help us minimize inbox volumes, we kindly ask that you not reply to this email simply to confirm you have resubmitted your revisions.

Kind regards,

Katalin Szabo
PLOS Neglected Tropical Diseases
plosntds@plos.org
